# Death Caused by the Injudicious Use of Antimony

**Published:** 1855-08

**Authors:** C. W. Le Boutillier


ARTICLE III.—*Death caused by the injudicious use of Antimony.* By C. W. Le Boutillier.

I was called on the 12th of June to visit Mr. James Cochran, who had then been under the care of a Quack (Z. Jodon) for three weeks.

He was employed in the pineries last winter, and has enjoyed very good health for the last ten years. About six weeks ago, while crossing the river, he fell in the water and got wet. In a day or two he was seized with a severe pain in the left hip, and in consequence of it had to come down in a wagon, (100 miles,) to receive medical aid. On his arrival here, (nine days after the accident,) he applied for treatment to the above named individual, who blistered him on the affected parts, and salivated him. He also gave him Tartarized Antimony, in the dose of 20 to 30 grs. per day, for three weeks in succession, with the exception of a few days that he omitted the medicine.

On his examination he also said that he had given the wine of Colchicum, in the dose of one tablespoonful, four times a day.

I found the patient as follows :—Age about 34 ; temperament sanguine—and learned that he had been a man of a powerful muscular frame, (weighing, two months ago, 206 lbs ,) but was then (at the visit) reduced to a squelete *vivante*, in fact so much emaciated that his friends did not recognize him on their arrival from above.

Symptoms.—Eyes sunken, and conjunctiva blanched; tongue coated with a brown fur, edge and tip very red deeply fissured on its median line, and perfectly dry. No difficulty existed in the protrusion of that organ, and its retraction seemed easy. Pulse. 120 in the minute and rather hard ; skin dry, shrivelled and of a deep yellow color.

Patient complains of great pain on pressure being made on the abdomen, the pain is more acute in the right iliac and epigastric regions. When aliments or liquids are taken into the stomach, they are immediately rejected. The nervous system has not been during his sickness in the least impaired, has had no cephalalgia, when interrogated answers without the least hesitation. Lungs and heart apparently normal. No derangement of the urinary organs—urine voided pretty copiously, and rather high colored.

Treatment.—Counter irritation on the abdomen ; give small quantities of acidulated ice water, and a small quantity of weak broth occasionally. On the 14th I called in Dr. Johnson, as counsel, and we continued the above treatment until the 17th, when the prostration became so great that we decided to use small doses of wine ; but we ultimately were obliged to give it in enemas, as the stomach would not tolerate it ; a ʒ of Spt. Turpentine was occasionally added to the injection. He had several dark and very offensive discharges from the bowels the first few days of our attendance, at the last they were of a better color and less offensive.

On the 20th Drs. Murphy and Anderson saw the patient, and approved of the mode of treatment.

Two days afterwards, the 22d, the irritability of the stomach was ameliorat d, and the pain in the abdomen was less severe ; but he gradually failed, and died between the hours of three and four on the night of the 24, retaining to the last his mental faculties.

Post Mortem Examination held fifteen hours after death by order of the Coroner, in the presence of Drs. Anderson, Ames, Murphy, Johnson, and LeBoutellier. (Jodon was also present.)

Body.—Emaciation very great. Skin.—Two large sores on sacrum, and one near the trochanter of the ischium—apparently bed sores. Bowels.—(View by raising the abdominal parietes.) Considerable congestion and inflammation generally; right iliac region very much so. Omentum inflamed, congested, and vessels enlarged. Stomach.—Dark appearance and inflamed on surface spleen a little enlarged, but healthy. Liver.—Healthy, gall bladder filled with darkish bile. Lungs and Heart.—Nothing abnormal. Bladder.—Normal and distended, with a pint and a half of urine. Alimentary Canal laid open; Stomach, whole surface inflamed. The mucous membrane was considerably thickened, softened, and disorganized in places, especially in its large curvature. Pylorus highly inflamed, with suppuration of its follicles. The bowels contained a small quantity of blackish thin matter, and a purpular eruption was found through their whole extent. Some of the pustules were well formed and defined, but the greatest number were in a state of ulceration. Duodenum, Jejunum, and Ileum.—Were found inflamed, mucous coat like stomach, and the surface of the bowels was copiously studded over with the eruption. A section six inches in length of the jejunum furnished six large patches of these agglomerated pustules, in a state of ulceration; they were generally covered over with a soft scab, and the muscular coat was not deeply involved. The cœcum was black and softened, very much like stomach. Colons.—Were found more or less highly inflamed, and the eruption as in small bowels. Kidneys.—Left healthy; but the cortical portion of the right one, the adjoining mesenteric glands, and two or three inches *of ascending colon were in a state of suppuration.*

After the autopsia the above named Physicians were sworn, and requested to answer separately the following questions. Q.—As a medical man, from your experience and from information gleaned from authors, in your opinion, what was the cause of the death of Mr. Cochran? A.—As far as we can judge from the history of the case, and the pathological lesions found in the bowels, we believe that he died from the injudicious use of Antimony. A number of other questions were made by the Jury and Coroner respecting medicines, their uses, and diseases, etc.

The Verdict of the Jury.

"That the said James Cochran came to his death by some cause unknown; but we think there is strong presumptive evidence that he came to his death by the improper use of tartarized Antimony, given by Z. Jodon."

Special Coroner, A. Leaming.
R. P. Upton,
W. F. Cahill,
J. B. Perkins,
J. C. McCain.
S. D. Hubbard.
L. G. Johnson.

